# Vaginal microbial profile of cervical cancer patients receiving chemoradiotherapy: the potential involvement of *Lactobacillus iners* in recurrence

**DOI:** 10.1186/s12967-024-05332-2

**Published:** 2024-06-17

**Authors:** Yichen Wang, Tingzhang Wang, Dingding Yan, Hongxia Zhao, Meixia Wang, Tingting Liu, Xiaoji Fan, Xiaoxian Xu

**Affiliations:** 1 Zhejiang Cancer Hospital, Hangzhou Institute of Medicine (HIM), Chinese Academy of Sciences, Hangzhou, Zhejiang 310022 China; 2 Key Laboratory of Microbial Technology and Bioinformatics of Zhejiang Province, Zhejiang Institute of Microbiology, Hangzhou, 310012 China; 3 Zhejiang Key Laboratory of Radiation Oncology, Hangzhou, Zhejiang 310022 China

**Keywords:** Vaginal microbiome, Chemoradiotherapy, Cervical cancer, Recurrence, *Lactobacillus iners*, Machine learning

## Abstract

The vaginal microbiome is an immune defense against reproductive diseases and can serve as an important biomarker for cervical cancer. However, the intrinsic relationship between the recurrence and the vaginal microbiome in patients with cervical cancer before and after concurrent chemoradiotherapy is poorly understood. Here, we analyzed 125 vaginal microbial profiles from a patient cohort of stage IB–IVB cervical cancer using 16S metagenomic sequencing and deciphered the microbial composition and functional characteristics of the recurrent and non-recurrent both before and after chemoradiotherapy. We demonstrated that the abundance of beneficial bacteria and stability of the microbial community in the vagina decreased in the recurrence group, implying the unique characteristics of the vaginal microbiome for recurrent cervical cancer. Moreover, using machine learning, we identified *Lactobacillus iners* as the most important biomarker, combined with age and other biomarkers (such as *Ndongobacter massiliensis*, *Corynebacterium pyruviciproducens* ATCC BAA-1742, and *Prevotella buccalis*), and could predict cancer recurrence phenotype before chemoradiotherapy. This study prospectively employed rigorous bioinformatics analysis and highlights the critical role of vaginal microbiota in post-treatment cervical cancer recurrence, identifying promising biomarkers with prognostic significance in the context of concurrent chemoradiotherapy for cervical cancer. The role of *L. iners* in determining chemoradiation resistance in cervical cancer warrants further detailed investigation. Our results expand our understanding of cervical cancer recurrence and help develop better strategies for prognosis prediction and personalized therapy.

**Supplementary Information:**

The online version contains supplementary material available at 10.1186/s12967-024-05332-2.

## Background

Cervical cancer is the fourth most frequently diagnosed reproductive malignancy among women worldwide [1]. In low development countries, cervical cancer ranks second in terms of morbidity and mortality among women, surpassed only by breast cancer, and most diagnosed patients are in advanced stages [2]. In China, data from the National Cancer Center showed approximately 111,000 new cases and 34,000 cancer-related deaths due to cervical cancer in 2019, indicating an existing significant threat to women’s health [3]. Currently, concurrent chemoradiotherapy (CCRT) is the recommended treatment for locally advanced cervical cancer and is implemented worldwide [4]. Owing to its significant advancements, CCRT greatly improves the survival rates of cervical cancer patients; however, more than a quarter of patients are challenged by distant metastasis and local recurrence [5]. Several studies have highlighted that the accurate prediction of cervical cancer recurrence, such as identifying reliable indicators, including International Federation of Gynecology and Obstetrics stage, radiomics features, HALP (Hemoglobin, Albumin, Lymphocyte and Platelet, HALP) score, and lymph node (LN) metastases, is crucial for deciding treatment strategies [6–8]. However, biomarkers for predicting cervical cancer recurrence remain largely unknown.

The vaginal microbiota plays a crucial role in maintaining the health of the female reproductive tract [9]. The microbiota that colonizes the female vagina is typically dominated by *Lactobacillus*, which produces lactic acid, H_2_O_2_, and other metabolites. This establishes an acidic vaginal environment and forms a physiological barrier on the vaginal surface [10, 11] that can restrict, coordinate, and balance with the host and environment to form a vaginal microecological environment and maintain normal vaginal microecological homeostasis [12–14]. The disruption of this balance may lead to bacterial vaginosis [15]. Several studies have highlighted that continuous disturbances in vaginal microecology are significant contributing factors to gynecological cancer [16, 17].

The microbiota has been extensively studied as an indicator for predicting treatment response in various diseases, including prostate cancer [18], lung cancer [19], inflammatory bowel disease [20], and hepatocellular carcinoma [21]. Additionally, microbial biomarkers are effective in predicting no responsiveness to immune checkpoint inhibitor administration [22]. Previously, we emphasized the role of *Lactobacillus* in differentiating the vaginal microbiome in human papillomavirus (HPV)-related cervical diseases, including cervical intraepithelial neoplasia and cancer [23]. However, the perturbation of the vaginal microbial community during CCRT and effectiveness of microbial biomarkers in predicting responsiveness to CCRT have not been thoroughly investigated.

Thus, we aimed to investigate the alterations in the vaginal microbial community after CCRT and determine whether microorganisms can serve as biomarkers to predict CCRT responsiveness. We collected 125 vaginal microbiome samples from patients with IB-IVB cervical cancer before and after CCRT, categorizing them into relapsing or non-relapsing post-treatment groups. By performing bacterial 16S rRNA amplification sequencing, we systematically analyzed variations in the diversity, composition, and function of the vaginal microbial community between patients in the relapsed and non-relapsed groups before and after CCRT. Additionally, machine learning was employed to identify keystone biomarkers in recurrent cervical cancer before CCRT. Collectively, this study aimed to explore alterations in the vaginal microbial community of patients receiving CCRT and with recurrence, which will provide pivotal evidence for the construction of a microbiota-dependent and non-invasive strategy for recurrence prediction and serve as basis for future studies in revealing the underlying mechanisms in cervical cancer recurrence.

## Methods

We prospectively used rigorous bioinformatics analysis to uncover the complex relationship between vaginal microbial composition and cervical cancer recurrence. And we basically followed the TRIPOD + AI statement for reporting multivariable prediction model development and validation.

### Sample collection and study design

A total of 125 samples from patients with IB–IVB cervical cancer who underwent CCRT at our hospital were enrolled in this study and all patients received standard-of-care treatment (CRT; 45 Gy of external beam radiation therapy with weekly concurrent cisplatin at 40 mg/m^2^ and brachytherapy). The vaginal microbiome was sampled using vaginal swabs as previously described [23], both before and after treatment. The swabs were placed in a sterile tube and stored at − 80 °C immediately. All patients were informed of the proposal for this experiment and signed an informed consent form. This study was approved by the by the Ethics Committee of Zhejiang Cancer Hospital (IBR-2021-42). In this study, the primary endpoint was progression-free-survival that the interval between the time when treatment started and the first recurrence of the disease. Recurrence of all patients, including both distant metastasis and local recurrence following initial treatment, were meticulously assessed through rigorous monitoring every 3 months during the first year following CCRT. We collected the physiological and biochemical index of patients, including age, HPV status, disease stage, hemoglobin, creatinine, albumin, C-reactive protein, cutaneous squamous cell carcinoma, syphilis, and hepatitis B, and the details of data can be obtained in the supplementary materials Dataset 1. Then, we developed a nomogram incorporating predictive factors (including vaginal microbiome and physiological and biochemical factors) for the prediction of recurrence and evaluated its efficacy.

### Bacterial genomic DNA extraction and 16S rRNA sequencing

We extracted DNA using PowerSoil® DNA Isolation Kit (MoBio) per the manufacturer’s instructions. The extracted DNA was separated on 1% agarose gel and quantified using a NanoDrop 2000 spectrophotometer. The final DNA concentration was diluted to 1 ng/µL. We amplified the V3 region of bacterial 16S rRNA using a universal primer pair and conducted polymerase chain reaction (PCR) cycles using Phusion® High-Fidelity PCR Master Mix (New England Biolabs). The amplification using the following PCR conditions: 94 ℃ for 3 min, followed by 35 cycles of 94 ℃ for 45 s, 50 ℃ for 60 s, 72 ℃ for 90 s; and finally 72 ℃ for 10 min. A 2% agarose gel was used to identify the PCR products and those ranging from 400 to 450 bp were collected for further use. We constructed DNA libraries using TruSeq® DNA PCR-Free Sample Preparation Kit (Illumina) per the manufacturer’s protocol and sequenced them on the Illumina MiSeq platform.

### Bioinformatics analysis

Raw data were merged using PEAR (PEAR 0.9.10 released) software after the barcode and primer sequence were removed, the obtained merging sequence was the raw tags. QIIME (v. 1.9.1) was used to filter the raw tags and remove sequences with more N or low quality to get high quality tags (clean data). Then, the UCHIME algorithm and gold database were used to remove chimera sequence and get effective tags. To learn the diversity of species composition of the samples, the effective tags were clustered into operational taxonomic units (OTUs) using uparse (v.8.1.1861) with 97% identify. Meanwhile, the most frequent sequences in OTUs were selected as the representative sequences of OTUs and annotated them by species using uclust method and SILVA ribosomal RNA database (https://www.arb-silva.de/). And then, the community composition of each sample was analyzed at each taxonomic level (kingdom, phylum, class, order, family, genus, and species). The abundance of each taxonomic level of each sample was also calculated based on annotation results.

### Statistical analysis

Principal coordinates analysis (PCoA) of the vaginal microbiota based on Bray-Curtis distance and alpha diversity (Shannon and Chao 1 index) analysis were performed using the vegan package (v 2.5-7) in RStudio. Network analysis at the genus level was performed based on a significant Spearman correlation matrix and visualized using the Gephi platform (v. 0.9.7). The analysis results and histograms of the community composition were visualized using GraphPad Prism (v. 8.0.2). The species-annotated (genus level) results of the OTU table were matched to the list of human probiotics in the probiotic database (https://probioticsdb.com/), and a heat map was constructed using TBtools. Microbial taxa abundance was compared between groups using the Two-sided *t*-test. Differences were considered statistically significant at *p* < 0.05. The schematic diagrams used in this study were constructed using the BioRender online platform (https://www.biorender.com/). The receiver operating characteristic (ROC) curves of the top five factors were established using the R pROC package, and the combined effects of these factors were calculated using the R tidyverse and Hmisc packages. The nomogram was constructed using the R rms package.

## Results

### Alterations in vaginal microbial community diversity in response to CCRT

**Fig. 1:**
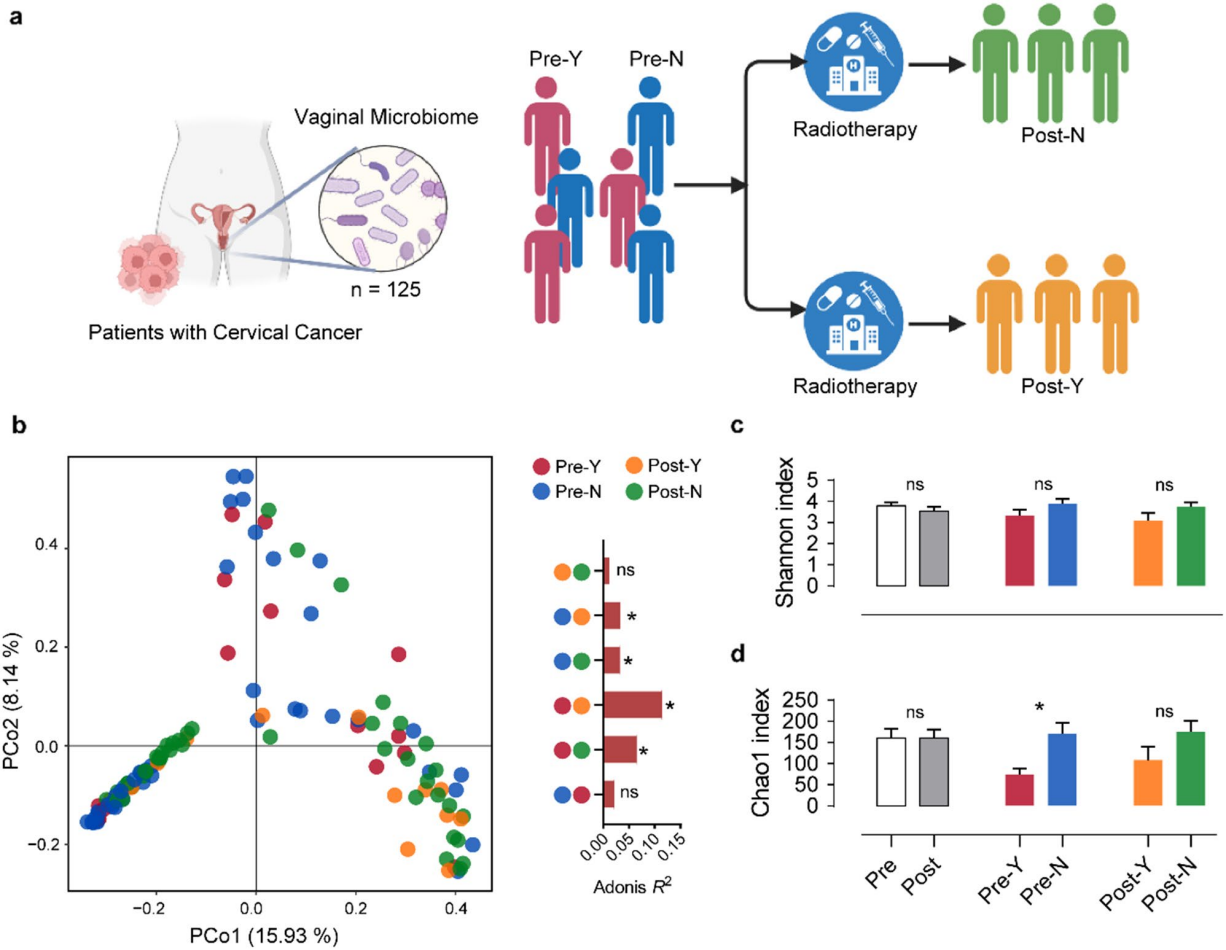
The vaginal microbial community diversity pre- and post-concurrent chemoradiotherapy (CCRT). (**a**) The overview of the size and classification of data in this study. “Pre-” and “Post-” represent the sample pre- and post-CCRT, respectively. “Y” and “N” represent vaginal microbiome samples from patients with recurrent cervical cancer or not, respectively. (**b**) The beta diversity (principal coordinates analysis) is based on the Bray–Curtis dissimilarity matrix of the vaginal microbial community. The asterisk represents the significant difference (Adonis *R*^*2*^ > 0.05, Adonis analysis) between the two groups, and “ns” represents no significant difference. (**c**) and (**d**) The alpha diversity (Shannon and Chao 1 indices) of the vaginal microbial community. The asterisk represents the significant difference (*p* < 0.05, Two-sided *t*-test) between the two groups, and “ns” represents no significant difference

A total of 125 vaginal microbial samples were collected from patients with cervical cancer before and after CCRT and further grouped according to whether recurrence occurred during the follow-up period (Fig. 1a). The vaginal microbial community structure was investigated by performing PCoA, and the results indicated a significant difference in the vaginal microbial community before and after CCRT (*p* < 0.05, Two-sided *t*-test), whereas cervical cancer recurrence had little effect on the microbial structure (Fig. 1b). The alpha diversity of the vaginal microbial community was further analyzed, and no significant difference was observed in the Shannon index between the different groups (Fig. 1c). Interestingly, the Chao1 index of the vaginal microbiome in pre-CCRT patients without recurrence were significantly higher (*p* < 0.05, Two-sided *t*-test) than those in patients with recurrence (Fig. 1d). Thus, this finding highlight that the importance of studying vaginal microbes before and after CCRT for cervical cancer.

### Alterations in vaginal microbial community composition in response to CCRT

**Fig. 2:**
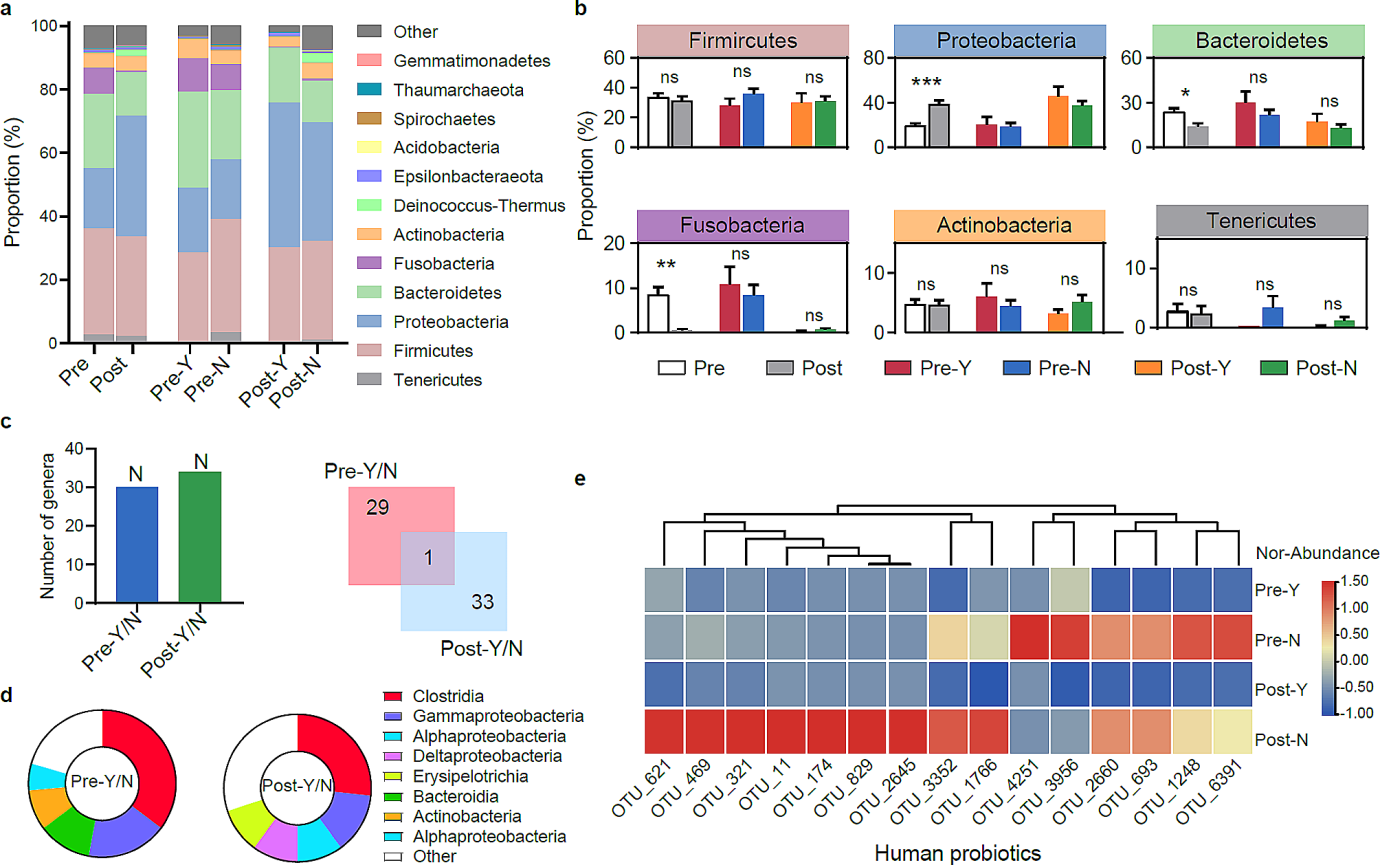
The vaginal microbial community composition pre- and post-concurrent chemoradiotherapy (CCRT**).** (**a**) The microbial community composition at the phylum level. (**b**) The relative abundance of six phyla in different groups. The asterisk represents the significant difference between the two groups (*, *p* < 0.05; **, *p* < 0.01; ***, *p* < 0.0001; Two-sided *t*-test), and “ns” represents no significant difference. (**c**) The number of genera with significant changes pre- and post-CCRT in the recurrent and non-recurrent groups. The square Venn diagram shows shared and special genera with significant changes in the pre- and post-CCRT groups. (**d**) The pie charts show the top 5 enriched Classes pre- and post-CCRT. (**e**) The abundance of human probiotics (OTU level) in four groups. “Pre-” and “Post-” represent the sample pre- and post-CCRT, respectively. “Y” and “N” represent vaginal microbiome samples from patients with recurrent cervical cancer or not, respectively

By performing bacterial 16S amplicon sequencing, we gained insights into the composition of vaginal microbial communities. *Firmicutes*, *Proteobacteria*, and *Bacteroidetes* were the most abundant phyla in the vaginal microbial communities both pre- and post-CCRT, accounting for more than 80% of the OTUs (Fig. 2a). A higher abundance of *Proteobacteria* was found after CCRT (*p* < 0.001, Two-sided *t*-test); however, *Bacteroidetes* and *Fusobacteria* abundance was much higher in the pre-CCRT cohort than in the post-cohort (*p* < 0.05, Two-sided *t*-test) (Fig. 2b). Moreover, cancer recurrence did not affect the abundance of dominant bacterial phyla in the vaginal microbiome. Additionally, the results showed that all genera with significant changes before and after CCRT were enriched only in the non-recurrence group, with little overlap between the pre- and post-CCRT groups (Fig. 2c). At the class level, the dominant taxa in the non-recurrent population were similar between the pre- and post-CCRT groups (Fig. 2d), and human probiotics dominated (Table S1; Fig. 2e). Alterations in the community composition indicated a significant decline in beneficial bacteria in the recurrent group, suggesting a disturbance in the vaginal microbial community.

### Alterations in vaginal microbial community functions and stability in response to CCRT

**Fig. 3:**
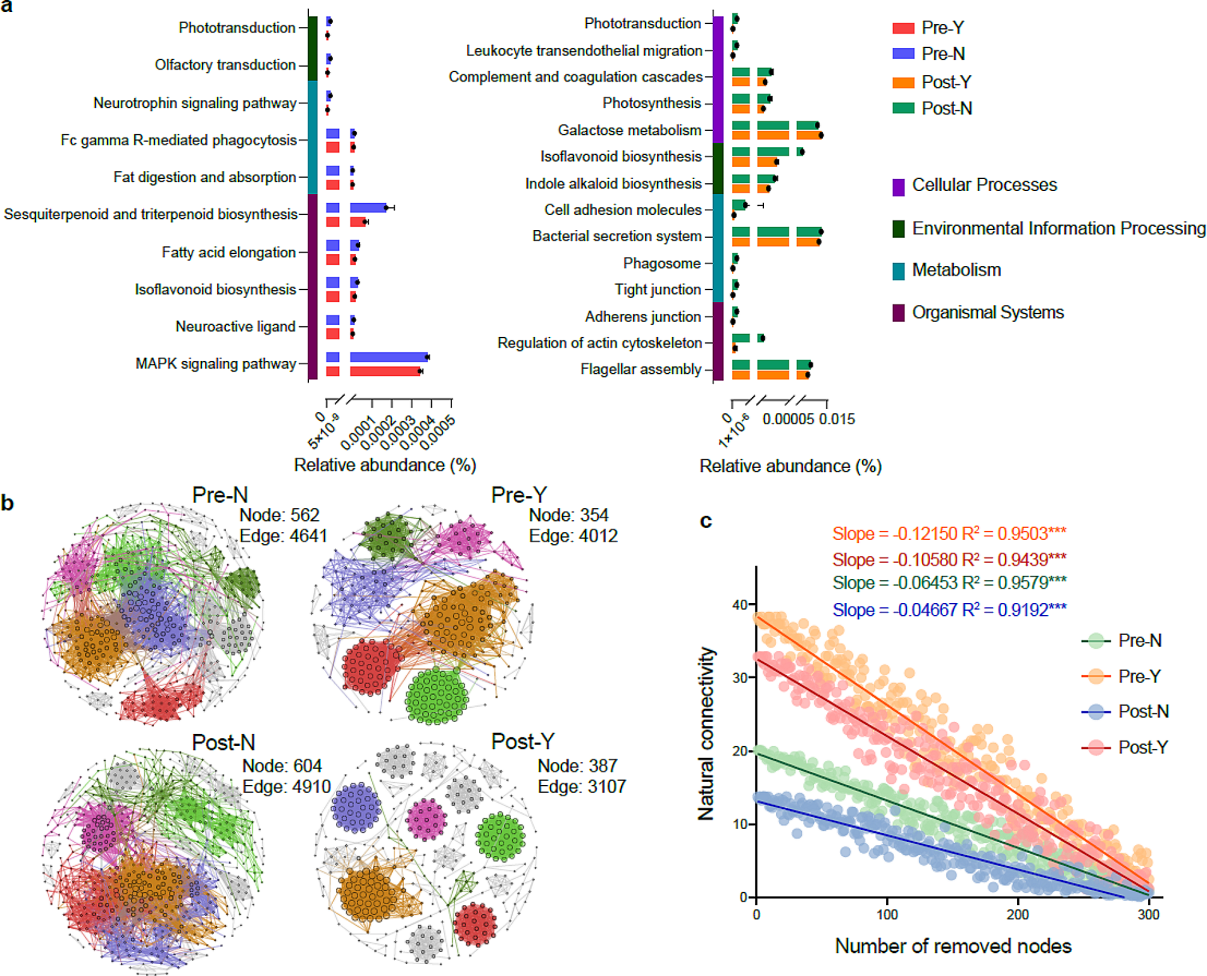
The vaginal microbial community function and stability pre- and post-concurrent chemoradiotherapy (CCRT). (**a**) The relative abundance of functional genes in recurrent and non-recurrent groups pre- and post-CCRT. All pathways have statistical significance (*p* < 0.05; Two-sided *t*-test). (**b**) The co-occurrence network is based on the Spearman correlation matrix of microbial interaction. Nodes are colored according to the module for clearer visualization, and node size represents the number of connections. (**c**) The robustness of four networks. The natural connectivity of the microbial network was calculated for every random node removed. Different colors correspond to different groups. “Pre-” and “Post-” represent the sample pre- and post-CCRT, respectively. “Y” and “N” represent vaginal microbiome samples from patients with recurrent cervical cancer or not, respectively

We further investigated whether alterations in the vaginal microbiome were effective in predicting recurrence after CCRT. Microbial function prediction analysis based on the Kyoto Encyclopedia of Genes and Genomes database showed that almost all functions were significantly reduced in the recurrent group before and after CCRT, including microbial lipid metabolism, immune signaling pathways, and other important functional pathways (Fig. 3a). Microbial co-occurrence network analysis showed that the number of nodes and edges in the microbial network in the recurrence group before and after CCRT was significantly lower than that in the non-recurrence group (Fig. 3b). Robustness analysis showed that the natural connectivity of microbial community after CCRT decreased faster (slope _(Pre−N)_ > slope _(Post−N)_ and slope _(Pre−Y)_ > slope _(Post−Y)_) with the removal of random nodes (Fig. 3c), indicating that CCRT significantly reduced the stability of the vaginal microbial community. Most importantly, the vaginal microbial community in the non-recurrence group (slope _(Pre−N)_ = -0.06453 and slope _(Post−N)_ = -0.04667) was significantly more stable than in the recurrence group (slope _(Pre−Y)_ = -0.1215 and slope _(Post−Y)_ = -0.1058). Thus, reduced microbial function and community stability further suggest a disturbance in the vaginal microbiome in the recurrence group (Y), implying a high risk of recurrence after CCRT.

### Identifying biomarkers of the vaginal microbiome in patients with and without recurrence pre- and post-CCRT

**Fig. 4:**
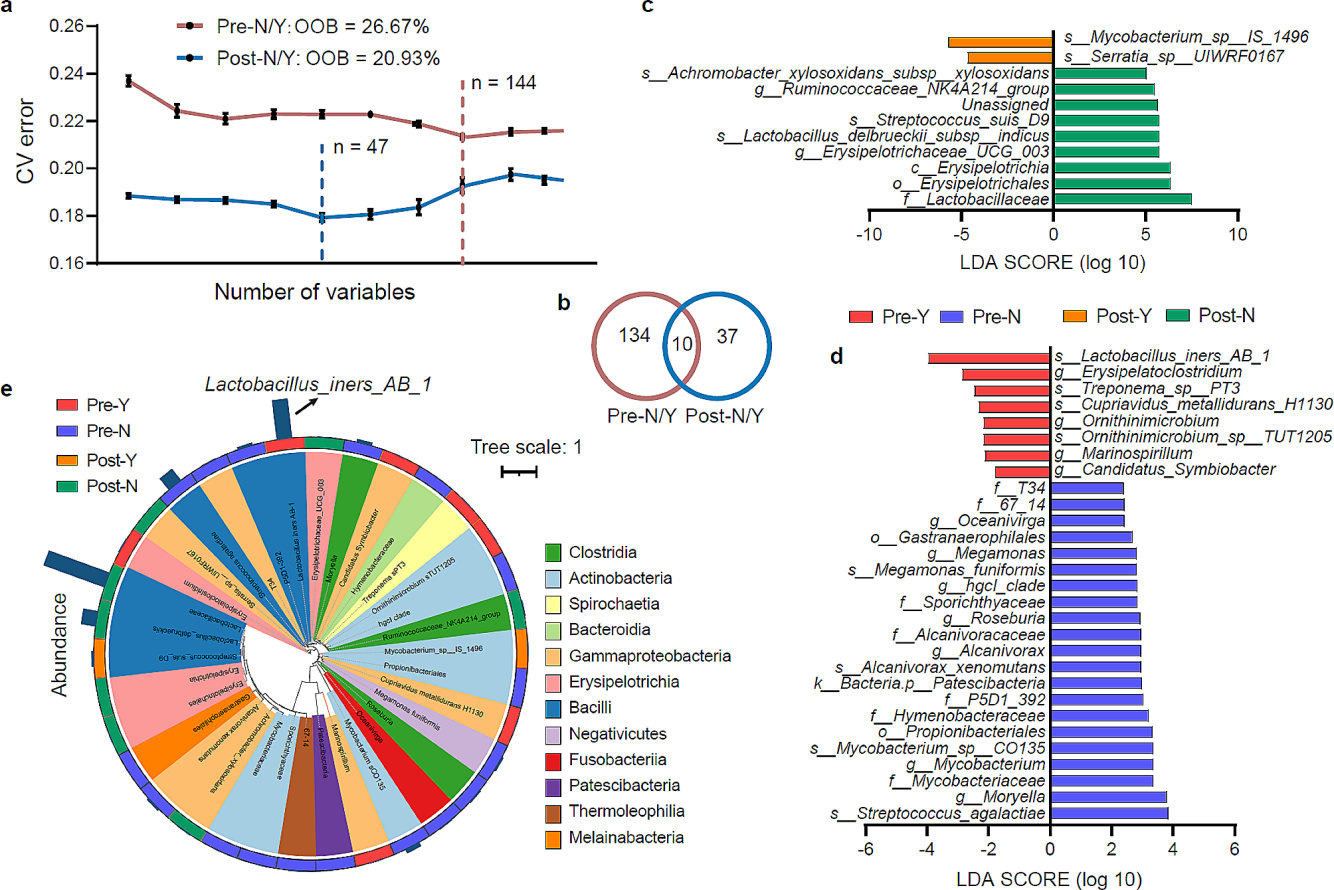
Identifying the biomarkers of cervical cancer recurrence pre- and post-concurrent chemoradiotherapy (CCRT). (**a**) Changes in tenfold cross-validation errors as the number of genera increases. The out-of-bag error was calculated by machine learning. “n” represents the number of genera with the minimum error. (**b**) The Venn diagram shows the shared and special biomarkers in the pre- and post-CCRT groups. (**c**) The bar chart mainly shows biomarkers whose absolute value of the linear discriminant analysis score is greater than the preset value (value = 4). (**d**) The bar chart mainly shows biomarkers whose absolute value of the LDA score is greater than the preset value (value = 2). Different color represents each group. The length of the bar represents the degree of the effect of biomarkers between different groups. (**e**) The cladogram shows the relative abundance of biomarkers across different groups

A binary classification model was constructed using the Random Forest algorithm in machine learning to identify biomarkers associated with cervical cancer recurrence before and after CCRT [24]. The Random Forest algorithm is a popular supervised machine learning algorithm used for both classification and regression tasks by constructing multiple decision trees during training. Based on the minimum error in the model, 144 and 47 biomarkers (species level) were identified in the pre- and post-CCRT groups, respectively (Fig. 4a). There were only 10 shared species of biomarkers between the pre- and post-CCRT groups, further suggesting the effect of CCRT on the vaginal microbial community (Fig. 4b). The linear discriminant analysis effect size further showed that in the recurrent group, *L*. *iners* and some human pathogenic bacteria were mainly enriched pre-CCRT, whereas harmful bacteria such as *Mycobacterium* and *Serratia* were mainly enriched post-CCRT (Fig. 4c and d). The phylogenetic relationships of these biomarkers were relatively diverse; Gammaproteobacteria, Actinobacteria, and Bacilli (class level) were dominant in the recurrent group (Fig. 4e). Notably, *L. iners* was most abundant in the recurrent group before CCRT. Based on these results, we speculate that *L. iners* may be an important predictor of cancer recurrence after CCRT.

### Identifying biomarker-driving phenotype factors in recurrent and non-recurrent patients pre- and post-CCRT

**Fig. 5:**
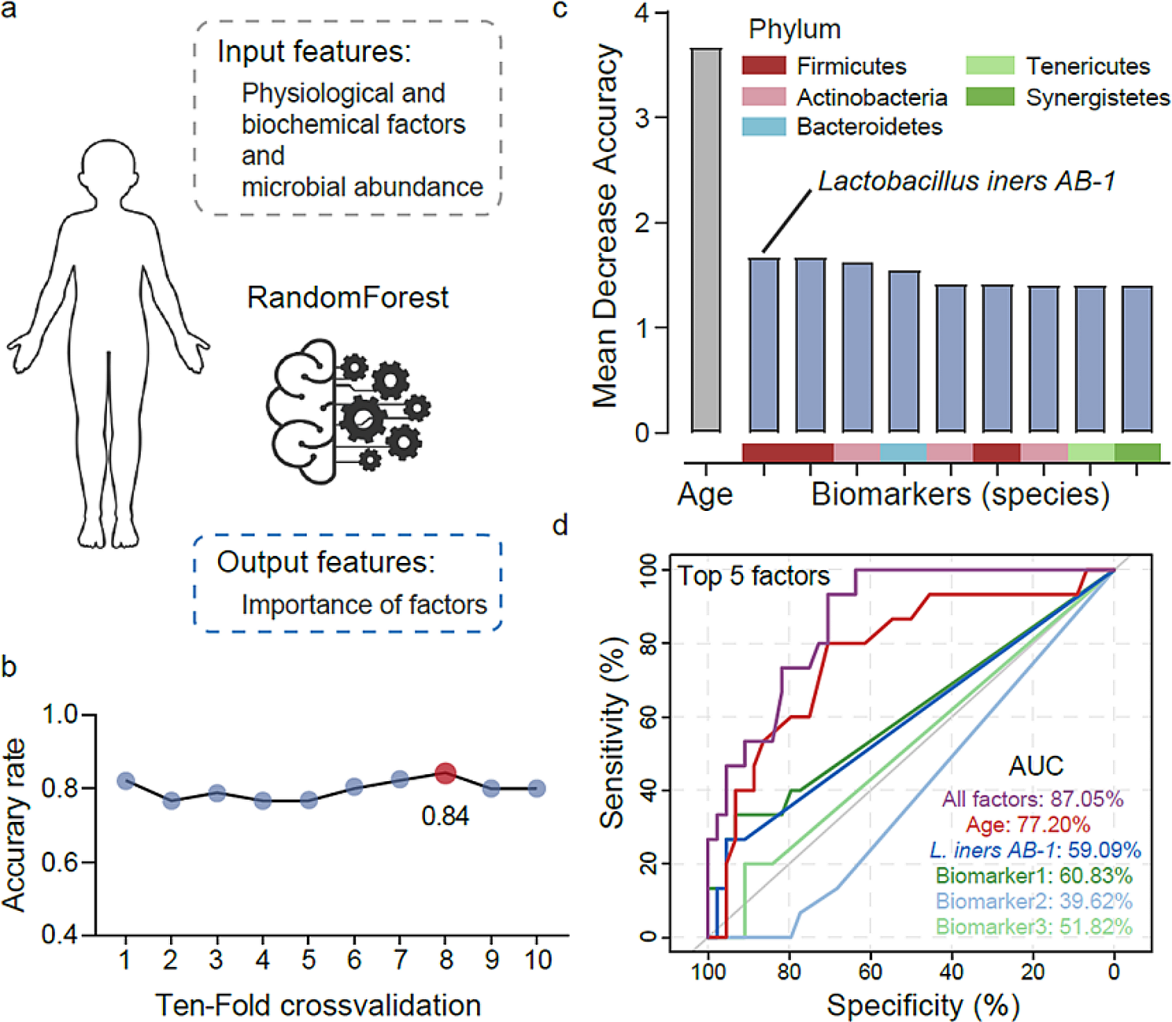
Identifying the phenotype factors of cervical cancer recurrence pre- and post-CCRT. (**a**) The schematic diagram introduces the basic information for constructing the random forest model. (**b**) The line chart shows the model with the highest accuracy based on ten-fold cross-validation. (**c**) The bar chart shows the importance value of various factors in the model. (**d**) The top five important factors were assessed using the receiver operating characteristic (ROC) curve. The area under the curve (AUC) was calculated to evaluate the performance of these factors. Biomarker 1: *Ndongobacter massiliensis* (Phylum: *Firmicutes*); Biomarker 2: *Corynebacterium pyruviciproducens* ATCC BAA-1742 (Phylum: *Actinobacteria*); Biomarker 3: *Prevotella buccalis* (Phylum: *Bacteroidetes*)

A classification model was constructed using machine learning analysis (Random Forest algorithm) to identify the important predictive factors (including microorganisms and physiological and biochemical factors) pre-CCRT (Fig. 5a). After selecting the optimal model (accuracy rate = 0.84) by ten-fold cross-validation to construct a classification model (Fig. 5b), it was found that the age of patients was the most important physiological factor in predicting recurrence pre-CCRT, and *L. iners* was identified as the most important microbial biomarker (Fig. 5c). The ROC curve of the top five factors indicated that four factors had predictive values, with area under the curve (AUC) values of 77.2%, 59.09%, 60.83%, and 51.82% (Fig. 5d). Among them, biomarker 2 (AUC < 0.5) along might not have a good predictive ability in this model. All five factors together had a best differential diagnostic ability, with an AUC of 87.05%. In addition to age, we calculated the importance of other physiological and biochemical factors, including HPV status, disease stage, hemoglobin, creatinine, albumin, C-reactive protein, cutaneous squamous cell carcinoma, syphilis, and hepatitis B, which ranked low based on their association with recurrence of cervical cancer (Figure S1). In addition, because of the uneven distribution of the number of samples between clusters (such as age) (Figure S2) and the large number of confounders, we did not stratify the confounding factors. For better diagnosis and prediction, a nomogram was constructed based on these five biomarkers using logistic regression analysis (Figure S3a). The ROC curve and AUC values were further evaluated to determine the diagnostic efficacy of the five biomarkers for recurrence (Figure S3b), and the performance evaluation of five factors in the nomogram were calculated (Table S3). Consequently, age, *L. iners*, and the other three biomarkers can be considered as key factors in predicting recurrence.

## Discussion

In this study, we investigated the vaginal microbiome of patients receiving CCRT and revealed the potential application of this microbiome in distinguishing cervical cancer relapse after CCRT.

The worldwide adoption of CCRT has made it a common treatment strategy for locally advanced cervical cancer [25, 26]. The advantage of CCRT is that chemotherapy and radiotherapy mutually enhanced the treatment efficacy leading to a synergistic effect [27], and thus improves the survival rate [28, 29]. But our findings revealed that CCRT markedly disturbed the stability of the vaginal microbiome, impaired its physiological functions, and negatively affected the vaginal health (Fig. 3). This effect may be attributed to the residual toxicity of platinum-based agents and the subsequent adverse side effects after CCRT [30]. Distant metastasis accounts for approximately 70% of cancer recurrences and is a primary contributor to mortality in cancer patients [31, 32]. Interestingly, we demonstrated that the vaginal microbiome of patients in the nonrecurrence group harbored a more stable community and abundant beneficial microbes than those in the recurrence group, both before and after CCRT, indicating an unhealthy vaginal environment (Fig. 2). These results are supported by recent investigations suggesting that medical interventions for cervical cancer may induce long-term changes in the microbiome and high biodiversity several years after treatment, thus weakening the immune system in the vagina [33]. These changes create favorable environmental conditions for the metastasis of tumor cells and the persistence of HPV [34].

Machine learning analysis further identified biomarkers corresponding to cancer recurrence, most of which were human pathogens, with *L. iners* being the most significant microbial predictor of recurrence in patients before CCRT (Fig. 4). Previous investigations consider *Lactobacillus* a beneficial bacterium in maintaining female vaginal health [35], whereas some investigations noted that *L. iners* behaves as a renegade in the vaginal microbiome [36, 37]. *L. iners* is strongly related to bacterial vaginitis, and its dominance in the vaginal microbial community indicates a high risk of pathogenic infections [38]. Additionally, *L. iners* is resistant to conventional antibiotics such as metronidazole, making bacterial vaginitis caused by *L. iners* challenging to cure with common treatment regimens [39, 40]. Furthermore, a recent study has revealed that *L. iners* can regulate resistance to CCRT in tumor cells, mainly through the production of L-lactate [41], an exchangeable metabolic coupling molecule between cancer cells, which relates to various treatment resistance mechanisms [42, 43]. Additionally, machine learning analysis identified patient age as a crucial recurrence factor from physiological and biochemical indices (Fig. 5). Patient age is commonly associated with HPV infection and menopause, and an age of 50 years is often regarded as the threshold for increased risk [44, 45]. However, the limitations in the size and diversity (e.g., age) of clinical samples hindered the exploration of correlation between specific phenotypes (e.g., elderly patients) and recurrence in this study. Overall, we may predict cervical cancer recurrence by combining microbial (*L. iners* and the three other important biomarkers) and patient’s age.

## Conclusion

In conclusion, considering current national development and medical standards, CCRT remains the optimal strategy for treating cervical cancer. However, the challenges followed with poor CCRT-related prognoses highlight the urgent need for developing improved treatment strategies for women with cervical cancer. Our study found that *L. iners* was involved in determining the recurrence rate after CCRT, warranting further investigation into its role and molecular mechanisms in cervical cancer recurrence. For patients at high risk of recurrence, we appeal to the clinicians to develop more aggressive treatment strategies such as adjuvant chemotherapy or selected immunotherapy, and conduct more intensive follow-up is also recommended. We highlight the importance of monitoring *L. iners* levels in vaginal microbiome samples in clinically treatments, especially in the older patients before CCRT. Ultimately, these insights provide valuable avenues for understanding cervical cancer recurrence and will help to develop better strategies for prognosis prediction and personalized therapy.

### Electronic supplementary material

Below is the link to the electronic supplementary material.

Supplementary Material 1

## Data Availability

The data used to support the findings of this study are available from the corresponding author upon request.
