# Supplementary material for: Vaginal microbial profile of cervical cancer patients receiving chemoradiotherapy: the potential involvement of Lactobacillus iners in recurrence
Source: J Transl Med. 2024 Jun 17;22:575. doi: 10.1186/s12967-024-05332-2 (PMC11184707; doi:10.1186/s12967-024-05332-2)
Supplement: Supplementary file 1 — Supplementary Material 1 [file 12967_2024_5332_MOESM1_ESM.docx]

**supplementary material**

**Vaginal microbial profile of cervical cancer patients receiving chemoradiotherapy: the potential involvement of *Lactobacillus iners* in recurrence**

Yichen Wang^1^, Tingzhang Wang^2^, Dingding Yan^1^, Hongxia Zhao^2^, Meixia Wang^2^, Tingting Liu^2^, Xiaoji Fan^2^, Xiaoxian Xu^1,3^ *

^1^ Zhejiang Cancer Hospital, Hangzhou Institute of Medicine (HIM), Chinese Academy of Sciences, Hangzhou, Zhejiang, China.

^2^ Key Laboratory of Microbial Technology and Bioinformatics of Zhejiang Province, Zhejiang Institute of Microbiology, Hangzhou, 310012, China.

^3^ Zhejiang Key Laboratory of Radiation Oncology, Hangzhou 310022, Zhejiang, China.

*Correspondence: Xiaoxian Xu, xuxx@zjcc.org.cn.


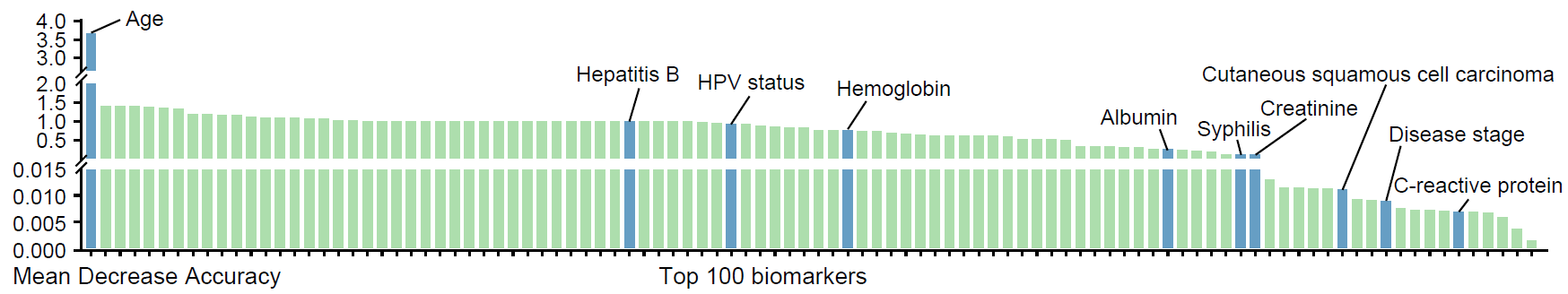


**Figure S1. The top 100 important biomarkers.** The ten physiological and biochemical factors rank based on mean decrease accuracy in our machine learning model.


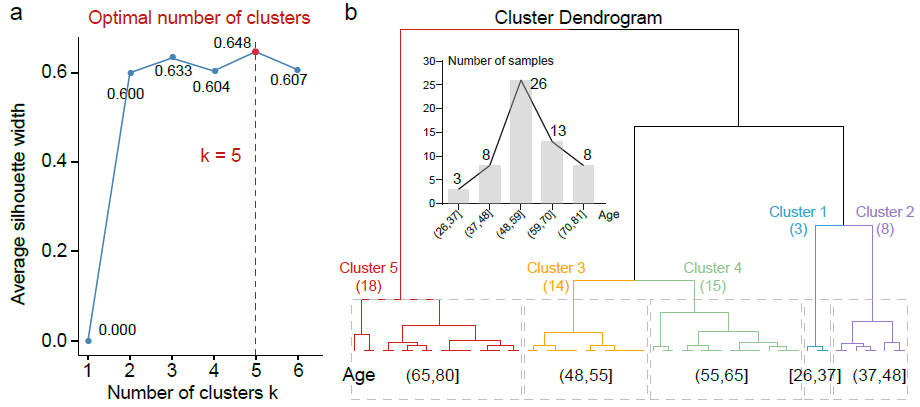


**Figure S2. The classification of samples based on patient’s age using k-means clustering algorithm.** (a) The optimal number of clusters (k value) based on k-means clustering algorithm. (b) The cluster dendrogram of samples. And the bar chart showed the distribution of samples among different age groups. Different colors represent different clusters.


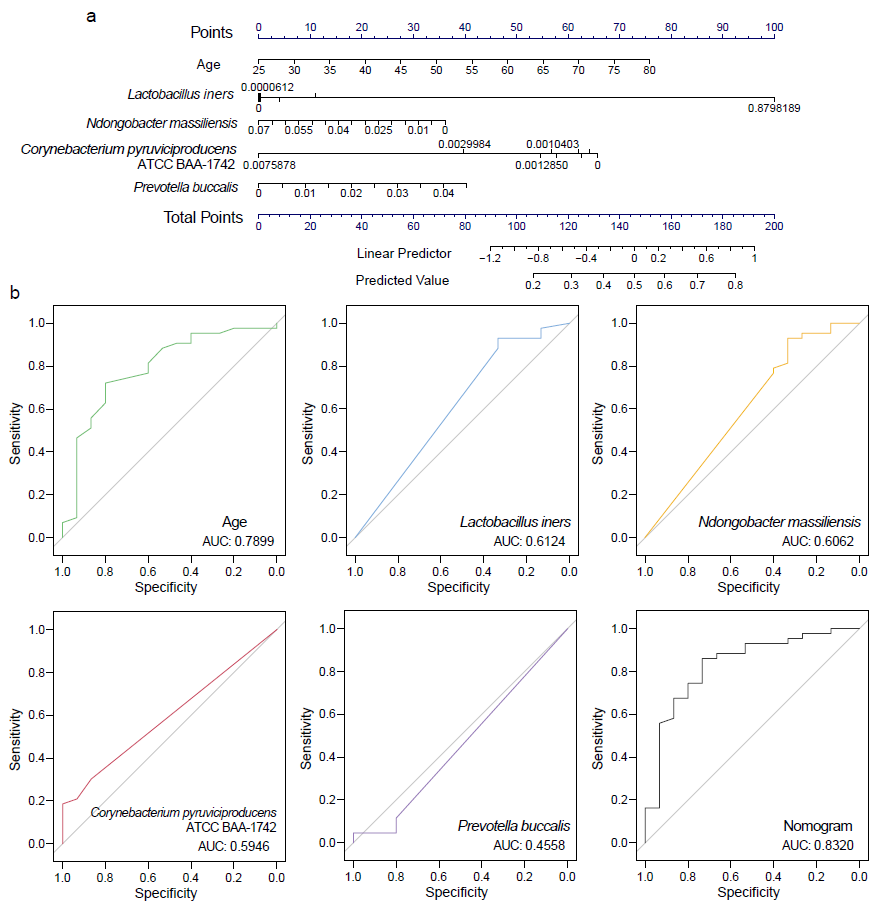


**Figure S3. The nomogram model of five biomarkers and diagnostic efficacy assessment.** (a) The nomogram constructed by five biomarkers. (b) The ROC curve and AUC value for the five factors and nomogram performance.

**Table S1. The abundance of human probiotics (OTU level) in four groups.** “Pre-” and “Post-” represent the sample pre- and post-CCRT, respectively. “Y” and “N” represent vaginal microbiome samples from patients with recurrent cervical cancer or not, respectively.

| **OTU ID** | **Probiotics for human (genus)** | **Pre-Y** | **Pre-N** | **Post-Y** | **Post-N** |
| --- | --- | --- | --- | --- | --- |
| OTU_11 | Lactobacillus | 0.000066 | 0.002011 | 0.000000 | 0.025348 |
| OTU_174 | Lactobacillus | 0.000000 | 0.000023 | 0.000000 | 0.001130 |
| OTU_321 | Lactobacillus | 0.000199 | 0.000294 | 0.000000 | 0.002056 |
| OTU_469 | Bacillus | 0.000000 | 0.000068 | 0.000000 | 0.000384 |
| OTU_621 | Lactobacillus | 0.000398 | 0.000361 | 0.000000 | 0.001965 |
| OTU_693 | Lactococcus | 0.000000 | 0.000023 | 0.000000 | 0.000023 |
| OTU_829 | Lactobacillus | 0.000000 | 0.000000 | 0.000000 | 0.000023 |
| OTU_1248 | Weissella | 0.000000 | 0.000158 | 0.000000 | 0.000090 |
| OTU_1766 | Lactobacillus | 0.000066 | 0.000136 | 0.000000 | 0.000294 |
| OTU_2645 | Clostridium sensu stricto 13 | 0.000000 | 0.000000 | 0.000000 | 0.000023 |
| OTU_2660 | Leuconostoc | 0.000000 | 0.000023 | 0.000000 | 0.000023 |
| OTU_3352 | Pseudomonas | 0.000000 | 0.000068 | 0.000000 | 0.000113 |
| OTU_3956 | Bacillus | 0.010205 | 0.026071 | 0.000000 | 0.005851 |
| OTU_4251 | Lactobacillus | 0.000000 | 0.000045 | 0.000000 | 0.000000 |
| OTU_6391 | Butyricicoccus | 0.000000 | 0.000045 | 0.000000 | 0.000023 |

**Table S2. The relative abundance of functional genes in four groups.** “Pre-” and “Post-” represent the sample pre- and post-CCRT, respectively. “Y” and “N” represent vaginal microbiome samples from patients with recurrent cervical cancer or not, respectively.

|  | **Pre-Y** | | | **Pre-N** | | |
| --- | --- | --- | --- | --- | --- | --- |
|  | Mean | SD | N | Mean | SD | N |
| MAPK signaling pathway | 0.00034119 | 0.00005290 | 15 | 0.00037784 | 0.00007040 | 44 |
| Neuroactive ligand | 0.00000142 | 0.00000139 | 15 | 0.00000712 | 0.00001680 | 44 |
| Isoflavonoid biosynthesis | 0.00001170 | 0.00000955 | 15 | 0.00002530 | 0.00003280 | 44 |
| Fatty acid elongation | 0.00001220 | 0.00000827 | 15 | 0.00002800 | 0.00004320 | 44 |
| Sesquiterpenoid and triterpenoid biosynthesis | 0.00006330 | 0.00005950 | 15 | 0.00016986 | 0.00027300 | 44 |
| Fat digestion and absorption | 0.00000047 | 0.00000061 | 15 | 0.00000117 | 0.00000175 | 44 |
| Fc gamma R-mediated phagocytosis | 0.00000628 | 0.00000518 | 15 | 0.00001010 | 0.00000790 | 44 |
| Neurotrophin signaling pathway | 0.00000000 | 0.00000000 | 15 | 0.00000000 | 0.00000000 | 44 |
| Olfactory transduction | 0.00000000 | 0.00000000 | 15 | 0.00000000 | 0.00000000 | 44 |
| Phototransduction | 0.00000000 | 0.00000000 | 15 | 0.00000000 | 0.00000000 | 44 |

|  | **Post-Y** | | | **Post-N** | | |
| --- | --- | --- | --- | --- | --- | --- |
|  | Mean | SD | N | Mean | SD | N |
| Flagellar assembly | 0.00344100 | 0.00203000 | 13 | 0.00507100 | 0.00287800 | 44 |
| Regulation of actin cytoskeleton | 0.00000017 | 0.00000036 | 13 | 0.00000109 | 0.00000250 | 44 |
| Adherens junction | 0.00000004 | 0.00000010 | 13 | 0.00000028 | 0.00000068 | 44 |
| Tight junction | 0.00000004 | 0.00000010 | 13 | 0.00000028 | 0.00000068 | 44 |
| Phagosome | 0.00000004 | 0.00000010 | 13 | 0.00000028 | 0.00000068 | 44 |
| Bacterial secretion system | 0.01013100 | 0.00157300 | 13 | 0.01148400 | 0.00239200 | 44 |
| Cell adhesion molecules | 0.00000011 | 0.00000026 | 13 | 0.00000079 | 0.00000182 | 44 |
| Indole alkaloid biosynthesis | 0.00001180 | 0.00001130 | 13 | 0.00002380 | 0.00003230 | 44 |
| Isoflavonoid biosynthesis | 0.00002720 | 0.00001660 | 13 | 0.00006300 | 0.00010400 | 44 |
| Galactose metabolism | 0.01147800 | 0.00329400 | 13 | 0.00931100 | 0.00226700 | 44 |
| Photosynthesis | 0.00000205 | 0.00000413 | 13 | 0.00001410 | 0.00002770 | 44 |
| Complement and coagulation cascades | 0.00000552 | 0.00000478 | 13 | 0.00001690 | 0.00002330 | 44 |
| Leukocyte transendothelial migration | 0.00000004 | 0.00000010 | 13 | 0.00000028 | 0.00000068 | 44 |
| Phototransduction | 0.00000004 | 0.00000010 | 13 | 0.00000029 | 0.00000068 | 44 |

**Table S3. Performance evaluation of five factors in the nomogram.**

| Cox Proportional Hazards Models | Sensitivity  (95% CL) | Specificity  (95% CL) | F1  (95% CL) | AUC |
| --- | --- | --- | --- | --- |
| Age | 0.956(0.924-0.987) | 0.985(0.972-0.998) | 0.974(0.961-0.987) | 0.7899 |
| *L. iners* | 0.921(0.877-0.965) | 0.953(0.917-0.989) | 0.852(0.821-0.883) | 0.6124 |
| *N. massiliensis* | 0.837(0.776-0.897) | 0.951(0.916-0.986) | 0.822(0.772-0.872) | 0.6062 |
| *C. pyruviciproducens* | 0.797(0.745-0.849) | 0.929(0.911-0.946) | 0.846(0.814-0.878) | 0.5946 |
| *P. buccalis* | 0.698(0.638-0.758) | 0.853(0.741-0.964) | 0.711(0.618-0.804) | 0.4558 |
